# Indoor Air Quality in Inpatient Environments: A Systematic Review on Factors that Influence Chemical Pollution in Inpatient Wards

**DOI:** 10.1155/2019/8358306

**Published:** 2019-02-27

**Authors:** Marco Gola, Gaetano Settimo, Stefano Capolongo

**Affiliations:** ^1^Department of Architecture, Built Environment and Construction Engineering (dept. ABC), Politecnico di Milano, Via G. Ponzio 31, 20133 Milan, Italy; ^2^Department of Environment and Health, Istituto Superiore di Sanità, Viale Regina Elena 299, 00161 Rome, Italy

## Abstract

**Introduction:**

Indoor air quality is one the main issues in which governments are focusing. In healing spaces, several research studies are reporting a growing number of data analysis and research works in order to guarantee and prevent health of users and workers. Currently the main investigations are about biological and physical risks; otherwise chemical ones are less investigated. Several countries are carrying out indoor air quality monitoring in those professional workplaces in which chemicals are used but also in some typically indoor (generic) spaces for the building hygiene assessment. The indoor air is affected by several factors that currently are analyzed punctually, without a whole scenario of all the variable performances. The authors have done a systematic review on the current state of the art and knowledge related to chemical pollution in healing spaces and the emerging strategies, supported by scientific literature, for healthy inpatient rooms and their indoor air.

**Methodology:**

The systematic review has been done through the analysis of papers from SCOPUS, DOAJ, and PubMed databases. The survey sample considered 483 scientific articles, between 1989 and 2017, and starting the systematic reading and analysis of the abstracts, only 187 scientific papers were selected, and only 96 were accessible.

**Discussion:**

Since scientific literature reports very different outputs and results, the resulting work from the survey is divided into specific fields of interest related to construction and finishing materials, installations, components, ventilation systems, processes, etc. Starting from the systematic reading, the paper classifies the factors of indoor air in four macroareas: outdoor air and microclimatic factors (temperature, relative humidity, air velocity, air change, etc.); management activities (management and maintenance activities, ventilation systems, HVAC, cleaning and disinfectant activities, etc.); design factors (room dimensions, furniture, finishing materials, etc.); and human presence and medical activities (users' presence, their health status, and medical activities carried out in inpatient rooms).

**Conclusion:**

The systematic review gives rise to a broad scenario on the existing knowledge regarding the indoor air pollution, design, and management strategies for healthy spaces and several emerging topics. Although the aim of the investigation is strictly related to chemical pollution, several considerations from the biological point of view have been listed. The systematic review, supported by the existing scientific literature, becomes a starting point for considering the importance of the topic and to stimulate the knowledge around this field of interest for improving studies, analysis, and simulations.

## 1. Introduction

Florence Nightingale [1] wrote in *Notes on Nursing*: *“the very first canon of nursing […] is […] to keep the air he breathes as pure as the external air, without chilling him [the patient].”* Healthcare facilities, as complex constructions, should be generator of health and well-being [2], and indoor air quality (IAQ) issue should be considered one of the most important topics to be assessed and monitored in healing spaces [3]. In fact, IAQ requires a great attention for guaranteeing healthy indoor air and protecting users, both patients and workers, against hospital acquired infections (HAIs) and occupational diseases [4, 5]. Through a proper control and improvement of IAQ, as well as disinfection of spaces and sterilization of medical instruments, hospital infections can be significantly prevented [6].

As the scientific community has revealed, the indoor air is a complex and dynamic issue in which physical factors, biological and chemical contaminants, generated in outdoor and indoor environments, can affect the health status of users.

It is well known that concentrations of pollutants in outdoor air are lower than in indoor one because internal sources, including HVAC systems, building materials, hospital staff, medical and human activities, etc., can highly influence the air performances [7]. In general, users may be exposed to a wide range of chemical compounds emitted from various products, such as disinfectants (ethylene oxide, glutaraldehyde, formaldehyde, alcohols, etc.), anesthetic gases, products for laboratories, or pharmacies [8].

In addition, in hospital wards, another influential pathogen source is related to the breathing of potentially infectious patients and medical procedure [9]; in fact, most of the patients spend most of their time in beds, while medical staff in a ward depend on their daily activities that they have to carry out.

Although in last decades, the indoor air obtained growing attention, the legislation and regulations in European scenario, related to limit values, are insufficient [10]. Currently, only hard hospital areas (such as sterilization, operating rooms, laboratories, etc.) have limit values of risk assessment such as the occupational exposure limit values (VLEP), the American Conference of Governmental Industrial Hygienists (ACGIH), or the threshold limit value (TLV) of the Scientific Committee on occupational exposure limits (SCOEL EU). Differently, for the other healthcare settings, as Settimo [10] highlighted, there are only limit values stated by World Health Organization guidelines, for ambient air and indoor air for generic environments, and values suggested by EU countries, which however are not specific for healthcare facilities.

More broadly, monitoring and analysis of chemical pollution by healthcare organizations lack as Gola et al. [11] empathized, unlike the biological contamination of which so far many scholars are advancing different considerations [12, 13].

From the chemical pollution's point of view, designers, managers, and hospital staff therefore have the strategic role on defining strategies for maximum infection control. In case of design mistakes, it becomes strategic to ensure proper management of the facilities, in particular with the HVAC system with constant air change, choosing furniture and building material with low volatile organic compound (VOC) emissions [14], regular and conscious maintenance, medical activities, and cleaning procedures (avoiding the use of sprays and other cleaning materials that generate VOCs, etc.) that take place inside the healing spaces [15, 16].

Starting from these considerations, a research group gave rise to a monitoring activity of air quality in inpatient rooms, focusing on chemical pollution for understanding the current values and room features, maintenance activities, and medical procedures [17]. For supporting the research project, the authors have done a systematic review on the current state of the art and knowledge related to chemical pollution in healing spaces and the emerging strategies, supported by scientific data, for healthy inpatient rooms and their indoor air. The focus of the investigation is strictly related to inpatient wards.

### 1.1. The Current Knowledge around Inpatient Wards

As Joseph and Rashid [18] sustained, the configuration of the inpatient room, as well as the design and health-related trends, is characterized by several factors, which can be divided into environmental (dimensional space and design features), management (relating to health procedures, cleaning and maintenance activities, etc. to be performed), and social (ensuring a humanized space for users) ones [19]. In particular, about the last one, Lyons and Petrucelli [20] stated that settings that incorporate healing features can improve users' health status and their safety, reduce stress, improve outcomes, and therefore shorten hospital stays, reduce the need for pain-killing drugs, reduce staff stress and fatigue, and increase the overall quality of health and effectiveness of its delivery [21]. Therefore, starting from the current knowledge, several impacts of environmental features on health and well-being in healthcare facilities can be subdivided into environmental safety, indoor air and thermohygrometric parameters, proper and efficient (natural or mechanical) ventilation, noise, finishing materials and furniture, lighting (natural), colors, ergonomics, accessibility, and wayfinding [19, 22, 23].

The articulation of inpatient wards is based on standard units of about 24–28 beds, equipped with all the necessary and main comforts. Each hospital ward has support services for medical activities and staff, such as a nursing station, the head nurse office, a kitchen, clean and dirty storages, material storage, public and staff restrooms, and ambulatories and doctor office.

**Figure 1:**
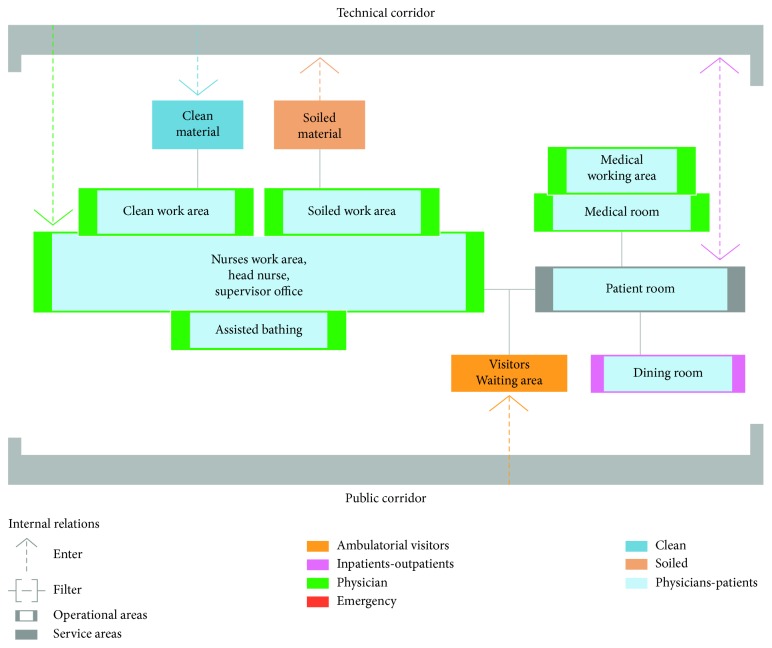
Functional organization of inpatient ward. Figure elaborated by the authors starting from AIA [24] and VVAA [25].

The functional organization is synthetized in Figure 1, and the distribution can be structured in a double-, triple-, or five-fold body.

Inpatient rooms can be subdivided into single or double bedroom. Several layouts have been studied, and it was defined that the area-per-bed of an average single bedroom is around 15 sqm. and 25 sqm. for a double bedroom.

Thermohygrometric parameters of healthcare facilities are defined by specific standards, which have evolved over the last decades. ASHRAE 170 [26] defines the following values:

- Temperature in hospital wards should be above 21°C and 24°C;
- Relative humidity in inpatient rooms, collective rooms and, if possible, in hallways should have a range between 40 and 60%;
- Air change: it must be able to achieve and maintain the air quality and movement conditions and the specific thermal and hygrometric conditions of the functional units (filtration, heating, cooling, humidification, and dehumidification). The values should be, for outdoor, 2 AC/h (air change rate minimum) and, totally, 6 AC/h, although Geshwiler et al. [27], starting from a literature review, suggested values between 3 and 12 AC/h related to the functional units;
- Ventilation system: the outdoor air flow rates for hospitals, clinics, nursing homes, etc., are 11 · 10^−3^ m^3^/s per person, excluding ambulatories and living rooms in which the values are around 8.5 · 10^−3^ m^3^/s per person and infectious rooms and operating rooms/birth rooms;
- Air velocity: should vary between 0.05 m/s and 0.25 m/s or no more than 0.30 m/s can be detected. In particular, the distinct speed for heating and cooling, in relation to the thermohygrometric design, clothing, and physical activities, so as to meet the well-being criteria, can be substantially identified with a range between 0.05 m/s and 0.20 m/s for heating and from 0.05 to 0.25 m/s for cooling;
- Air filtration: classification in fourteen filter classes, for different types of wards and services, these range from a minimum of 6 to a maximum of 12, however, with a filtration efficiency of at least *M* + *A* (medium-high);
- Pressure: typically, the leakage area in a room is set around 0.03 m^2^, usually with doors undercut by about 1–1.5 cm; undercutting minimizes resuspension due to the door scraping the floor. The minimum pressure difference between rooms and corridors and rooms and toilets is usually about 2.5 Pa [28].

On the outdoor side, as ASHRAE [29] suggested, the correct location of the outdoor air intakes and exhaust outlets of ventilation (HVAC) systems should include specific precautions. Indoor air quality depends mostly on the supply. Outdoor intakes should be localized as far as practical, but not less than 9 m from cooling towers, ventilation exhaust outlets from hospital or adjacent buildings, combustion equipment stack exhaust outlets, plumbing vent stacks and medical-surgical vacuum systems, and areas that may collect vehicular exhaust and other noxious fumes [30]. In addition as American Society for Healthcare Engineering highlighted, outdoor air intakes should protect and reduce accessibility to the animals (birds are a major source of *Aspergillus*, while insects provide waste matter that is both a culture medium for pathogens and irritating to humans, etc.) and yet allow for cleaning [31].

**Figure 2:**
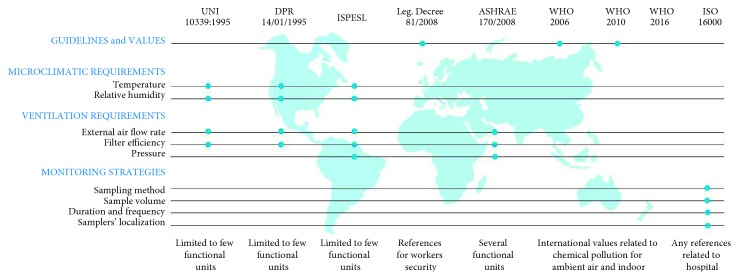
General overview of the existing norms and guidelines related to indoor air.

Among the requirements to be guaranteed in inpatient wards, it is necessary to respond to requirements by regulations related to finishing materials (in terms of hygienic conditions). The norms, in fact, define the performances that given materials should have in order to be used in specific environmental units (Figure 2 and Table 1).

In addition, as local health rules and building regulations require, it is necessary to guarantee a minimum height (around 2.7 and 2.8 m) and an adequate ventilation and lighting ratio (typically 1/8; [32], although the air can be replaced by ventilation system, as in hotel facilities).

In conclusion, regarding the maintenance and the efficiency of spaces, some countries have stated some limitations regarding cleaning products. In particular in Italy, Ministerial Decree (18/10/2016, number 262) states the adoption of the minimum environmental criteria for the assignment of the sanitization service for the sanitary facilities and for the supply of detergent products.

## 2. Methodology

A systematic review has been done through the analysis of papers taken from three main databases: SCOPUS, DOAJ, and PubMed. The first ones, SCOPUS and DOAJ, collect publications from various disciplines and in particular, for the purposes of the investigation, from chemistry, engineering, architecture, and medicine fields, while PubMed from the healthcare, medicine, and biological fields.

In order to facilitate the research, since the selection of some specific terms did not permit to reach an adequate sample of identified articles, several searches were made to access a rather significant number of papers.

In addition, since the issue is a nonwidespread topic, the choice of using few terms has been functional for the purpose of the research with several proceeding papers and articles with very different topics and outputs.

**Figure 3:**
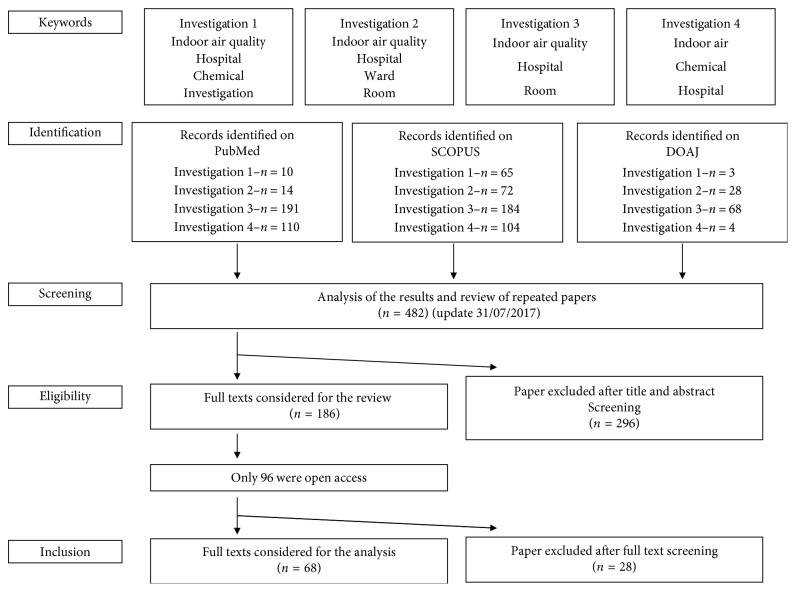
Systematic review organization.

Among the data analysis, there are some articles that are common and, in general, as Figure 3 shows, the survey sample analyzed 483 scientific articles, between 1989 and 2017 (the survey was updated until July 2017). From the systematic reading and analysis of the abstracts, only 187 scientific papers were selected. Among these, only 96 (51.33%) were accessible (Open Access, Research Gate, Mendeley, etc.) and considered in the systematic review.

Since scientific literature reports very different outputs and results, the resulting work from the survey is divided into specific fields of interest related to construction and finishing materials, installations, components, ventilation systems, process modes, etc.

## 3. Discussion

### 3.1. A General Overview of the Systematic Review

The focus of the systematic review is related to a specific functional unit of the hospital in which vulnerable users stay for several days and workers operate all year, and the indoor environmental quality (IEQ) is fundamental. The review gives rise to three main fields of interest: monitoring activities in real case studies, simulations of factors that affect the indoor air in theoretical case studies, and studies related to punctual aspects (cleaning products, finishing materials, HVAC, etc.).

For the purpose of the investigation and trends related to design and management strategies, the systematic review excluded the monitoring activities; in fact, as Gola et al. [11] empathized, although there are some monitoring experiences, their data are incomparable because their methodology, aims and scope, and data analysis are very different from each others for (a) their detectors and monitoring methods; (b) their climatic conditions, sources and outdoor pollution, healthcare activities and products used, environment dimensions and configuration of the room, etc.; (c) chemical compound analysis related to different healthcare issues investigated by the healthcare organizations, and the variable of time.

In addition, their outputs do not investigate the room configurations and the factors that affect the air quality. In any case, the representative case studies have been analyzed punctually in the discussion, for supporting the emerging considerations from the systematic review.

**Figure 4:**
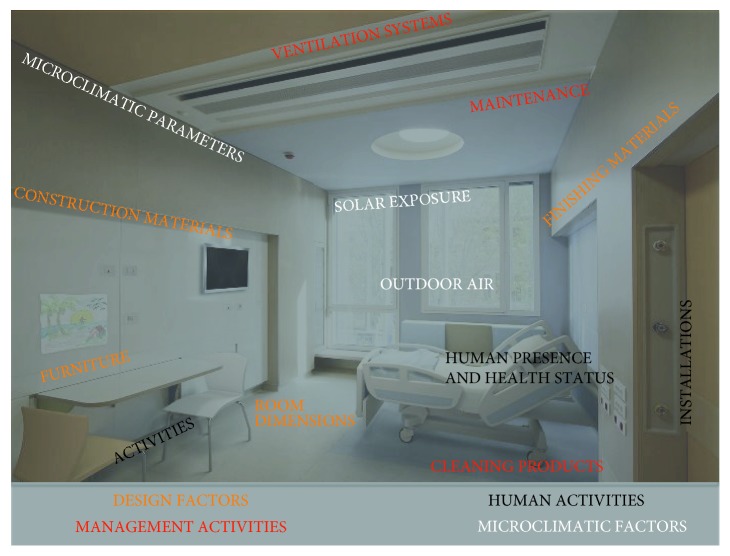
Factors that affect IAQ in inpatient room.

Therefore, starting from the systematic reading, it is possible to classify the factors of indoor air in four macroareas, as Table 2 and Figure 4 emphasize.

Although the systematic review aims to define the current trends in design and management strategies for healthy inpatient spaces, the complexity of the topic and the many factors involved often do not allow to define universal solutions. This is the reason why in the next paragraphs the topics give rise to several considerations and suggestions. For supporting the contents, the authors refer also to grey literature on the topic.

The analysis of the paper is related to chemical pollution and VOC emissions during the time; the emerging issue related to chemical pollution is a health risk for all the users. For this reason the systematic review considers the users both as the hospital staff and the patients, without any differences between occupational and patient safety.

## 4. Outdoor and Microclimatic Factors

### 4.1. Outdoor Issues and Site Localization

In architectures for health, local outdoor sources of air pollution represent a potential threat to IAQ. In fact, a contribution from outdoor sources to indoor air may come from the general outdoor air pollutants determined by sources and long-range transport, depending on wind direction [33], but also from nearby sources of air pollution such as road traffic, including emissions by parking facilities [34].

Although the indoor values are worse than outdoors, several research works have demonstrated that indoor pollution in inpatient rooms is highly influenced by outdoor air. Pereira et al. [35] demonstrated that the influence of outdoor air was clearly observed during the night in unoccupied inpatient rooms with an apparent lack of significant indoor particle production. It is possible that the higher total and fluorescent particle concentrations might reflect the timing of sampling which would result in differences in outdoor air change through window openings and air handling units (AHUs).

As Oppio et al. [36] highlighted in a research project related to hospital localization, the main environmental criteria related to site selection should consider (a) noise and air pollution and (b) the presence of unhealthy industries in the neighbourhood. In particular, regarding air pollution, referring to regulations, three different pollutants should be considered, as the WHO [37] states: (a) particulate matters (PM_10_ and PM_2.5_), daily and annual limits; (b) ozone (O_3_), respecting the limits for the protection of human health; (c) nitric oxide (NO), daily and annual limits [38].

In addition, industries, as manufactures or factories that produce vapours, gases, or other unhealthy fumes that are dangerous to the health of the inhabitants and users, should be far of a minimum radius from the boundary of hospital site, as defined by local regulations, although they are already updated [32].

### 4.2. Microclimatic Parameters

The systematic review highlights the role of microclimatic factors. Hospital air quality indices include typically temperature and humidity, levels of carbon monoxide (CO) and carbon dioxide (CO_2_), total volatile organic compounds (TVOCs) (a brief note related to total volatile organic compounds (TVOCs): they are, in general, a group of compounds not generally used (refer to ISO 16000); TVOC is a group of a wide range of organic chemical compounds and it is a general term for listing several compounds in ambient air each country and authority list different compounds, and therefore data analysis is not always comparable), particulate matters (PM), bacteria, etc.

Both temperature and relative humidity are recommended indices to reflect the efficiency of ventilation systems in healing spaces; however, they do not provide information on how separate parameters, such as air change rate and increase of surface area by the furniture in environmental units, may affect the deposition of particles and, therefore, the relationship between the distribution of aerosol particles and pollutants in the room [39].

As several authors demonstrated, patients in climate-controlled environments generally have a faster and more effective physical improvement than in uncontrolled ones [40], and, as a consequence, temperature and relative humidity can inhibit or affect the growth of microorganisms and VOC emissions [41].

### 4.3. Solar Exposure

The scientific community has highlighted, supported by Ulrich's studies and evidence-based design (EBD) demonstrations [21], in last decades, the importance of natural lighting and the relationship with external view for patient's (psychological and physical) well-being [42].

In addition, it is well known that direct sunlight is a powerful germ-killing agent, and daylight is more powerful than artificial light in suppressing streptococcal and respiratory tract infections [43, 44].

Designers should consider, in relation to the solar exposure, technological solutions (façade technologies, glass selection, passive strategies, etc.) for controlling the overheating of inpatient rooms [45]. It is well known, in fact, that overheating can cause discomfort for users, but in the same time, increasing of microclimatic parameters causes potential emission growth of substances by finishing and furnishing materials.

Jung et al. [46] demonstrated how the IAQ varies with ventilation types, especially with window opening, and working areas in hospitals. Data analysis highlighted a better correlation between indoor and outdoor PM_2.5_ concentrations in facilities with window openings than in those with only air conditioning. On the contrary, higher levels of bacteria were measured in healthcare environments with window openings, and the ratios of indoor and outdoor fungal concentrations were higher demonstrating that outdoor air significantly affects indoor fungal levels.

## 5. Management Activities

### 5.1. Cleaning and Maintenance Activities

Cleaning is the process of identifying, removing, and properly disposing of contaminants from a surface or environment, as Salonen et al. [23] defined. Disinfectant activities are crucial even if source management, management activity, design intervention, and dilution ventilation have all been used optimally to control infectious aerosols [47].

Inpatient ward is considered a low/medium-risk functional area. Therefore cleaning of the room should be done at the beginning of the day and, another time, in the afternoon. Extraordinary cleaning, in case of emergency and/or specific therapies, should not be excluded.

As several authors highlighted, cleaning and disinfectant activities of floors, walls, furniture, and equipment can increase humidity and, as a consequence, it can facilitate growth and survival of microorganisms.

Nowadays, many hospitals use cleaning solutions and detergents for reducing the risk of infection, thus, increasing the levels of TVOCs [48]. In fact, as several scholars have observed, airborne exposures from cleaning and disinfectant products are challenging to quantity because they are mixtures of ingredients having a range of volatilities and other physicochemical properties and thus require multiple monitoring techniques [49–51]. An emblematic case study by Llamosas et al. [52] highlighted the inadequacy of air quality in a dialysis center: an inadequate ventilation system, with the incorrect use of cleaning products affected highly the performances of indoor air.

In healthcare organization, the purchases of detergents, disinfectants and hydroalcoholic solutions are managed through a tender (for many functional units of the hospitals), although the variety is relatively limited. An investigation around the chemical composition of cleaning products by Berrubè et al. [53] identified 112 commercial products and 125 distinct substances used in French hospitals. The analysis listed 16 detergents and disinfectants, 4 hydroalcoholic solutions, and only 25 medicines and antiseptics for inpatient wards (they usually consist of several substances, the number of substances is about twice that commercial products). The analysis emphasized the products for inpatient room because, in the departments investigated, the drugs administered in aerosol form are mainly prepared directly at the patient's bed [53].

Another research project by Bello et al. [49] identified the cleaning and disinfectant products used for common cleaning activities in six hospitals in Massachusetts in United States. A set of frequently used products was selected for further quantitative exposure characterization. Selection criteria specified considered the following: (a) one volatile ingredient identified, at least, as a potential respiratory hazard based on previous qualitative assessment, (b) being task specific, and (c) being available via commonly used distributers. The analysis of material safety data sheets observed that 2-buthoxyethanol, ethanolamine, ethanol, ethylene glycol, and propylene glycol mono-ethyl ether were the main components in all of the products selected, with a range concentration between 0.5% and 10%.

Another specific investigation on the effects of detergents in healthcare facilities was done by Bello et al. [50]. They have developed some simulations of cleaning tasks in some bathrooms in different hospitals, already, in Massachusetts. The aim of the investigations was to control task frequency, duration, and environmental conditions. To investigate the feasibility of analyzing a wide range of airborne concentrations, cleaning tasks were simulated in different conditions (room's volume, ventilation system, concentrations of the volatile components in the products, etc.). The simulations showed TVOC concentrations steadily increasing with time during task performance, reaching the peak at the end of the cleaning activities. TVOC concentrations after the tasks declined exponentially to the previous concentrations in 20 minutes [50].

The systematic review revealed some specific case studies and suggestions for good practices. In particular, in South Korea, many types of disinfectants have been widely applied in humidifiers to prevent microbial contamination for some decades, but their use has been banned since 2011 due to concerns about their health effects, as Korean Society of Environmental Health reported [54]. The investigation evidenced that the use of humidifier peaks during the winter and spring seasons in healing environments with more than 70% in atopic dermatitis patients [55]. Several epidemiological analyses in South Korea, in fact, have highlighted that humidifier disinfectants can cause lung diseases, widespread lung fibrosis, interstitial pneumonitis, etc., necessitating lung transplantation [56].

The scientific community has also investigated the role of vacuum cleaners. Although currently, there are some vacuum cleaners with high-efficiency particulate-arrest (HEPA) filters and synthetic or double-bag collection systems, some authors, such as Clark et al. [57]; demonstrated an increase in airborne bacteria during cleaning procedures. In fact, as Veillette et al. [58] demonstrated that vacuum bags can accumulate bacteria, molds, endotoxins, and allergens.

As a final consideration, the cleaning activities should be done regularly and accurately. Irregular cleaning and floor sweeping cause the continuous deposition of particles. As Gulshan et al. [59] sustained, if the cleaning activities decrease over time, particulate matter levels increase considerably; the deposited dust is resuspended due to movement of people, as well-evidenced by Tormo-Molina et al. [60], as there is no removal process.

Airborne particulate matter, biochemical aerosol and dust concentrations are usually higher during the cleaning and disinfectant activities, although cleaning procedures are generally effective in reducing (but not eliminating) microbial contamination [30]. Thus, inpatients should not stay in hospital areas during the cleaning procedures and, as Ahmad et al. [61] stated, the cleaning staff should wear protective suits and masks to protect themselves from biochemical contaminated air.

### 5.2. Ventilation Systems

Building ventilation systems have the role to provide adequate physical conditions and perceived air quality to users through fresh air supply, heat removal, and pollutant dilutions. In architectures for health, they should prevent cross infection risks, harmful emissions, and pathogens' spreading. According to Friberg et al. [62], a good design of the ventilation system can decrease the infection risks.

As La Mura and Merici [63] suggested, ventilation systems in healing environments aim to achieve air quality and thermohygrometric conditions for guaranteeing users' prevention from contaminants and indoor pollution, obtaining adequate and comfortable indoor spaces and, in addition, for permitting management processes for carrying out the activities and for the operation for diagnostic and therapeutic therapies.

As several scholars state, the achievement and management of these conditions are highly related both to design strategies (such as layouts and distribution systems, finishing and construction materials, medical devices and equipment, etc.) and management aspects (such as products, cleaning and maintenance activities, etc.).

Ventilation for hospitals is very hard: ventilation influences several spaces and processes, many of them with very specific requirements. There are several approaches that can be subdivided into: (a) natural ventilation versus mechanical and hybrid ventilation; (b) outdoor air only (without any recirculation) versus secondary/recirculation air systems; (c) ventilation rate per person (or per emission source) versus air changes per hour (ACH); (d) concentration limits for particles (particles/m^3^) and/or for microbiological contaminants (CFU/m^3^) [64].

Mechanical and natural ventilation has two different principles: mechanical ventilation guarantees all room air distribution (constant and regular), while natural ventilation generates a room air distribution that can either be described as mixing flow or as displacement flow (inconstant and irregular [65]). Mixing flow occurs if the temperature difference is highly reduced and the flow rate is large; differently, a high temperature difference and a small flow rate generate displacement flow.

From the environmental point of view, natural ventilation is considered as a healthier ventilation strategy, which is driven by natural forces of wind-driven pressure difference and temperature difference through windows and door openings, etc.

If indoor spaces are greater and deeper than 6 m from the façade, mechanical ventilation generally is required with 100% fresh air system, presumably avoiding the recirculation of airborne pathogens [66]. As Zuraimi et al. [67] observed, there were higher ratios of indoor and outdoor PM_2.5_ and ozone in childcare centers with natural ventilation than those with mechanical one.

It is suggested as a low-cost and energy-saving alternative, but it is necessary to define the hospital building's target, ambient wind conditions, the surrounding buildings and obstacles, etc. [68], as Mohammed et al. [69] have developed in their country.

On the contrary, natural ventilation, such as that one provided by opening windows, is not considered totally an adequate method for infection control by many ventilation standards and guidelines; in particular, a guideline recommends that natural ventilation systems should achieve specific ventilation rates that are significantly higher than the ventilation rates required in practice guidelines for mechanical systems [70].

Differently, mechanical ventilation includes central air conditioning (such as AHU, fan cooling unit: FCU, and AHU mix FCU) and noncentral air conditioning (window type, single-split type, etc.). Central air conditioning with filtration system can be used to remove outdoor air pollutants [46]. Their design should permit accurate control of environmental conditions, with low maintenance costs, to facilitate the technical operations of cleaning and replacement of parts, energetically sustainable and eco-friendly, and which allows to contain the air flows' in and out between the various areas [29, 30].

Different air distribution systems permit several configurations for users' protection from airborne pollutants. Their design considers, in general, source control, particulate filtration, moisture control, dilution, air diffusion, and air-pressure control. These steps permit to reduce airborne contaminants and pathogen concentrations from the hospital room [64].

The pollutants are almost fully mixed in a room ventilated by mixing or vertical ventilation when it is totally occupied [65]. As Śmiełowska et al. [71] stated, the occurrence of BTEX (benzene, toluene, ethylbenzene, and xylene) and acetaldehyde compounds in hospital rooms is strictly connected with the interaction with the atmospheric air as a result of ventilation systems [72–74].

As Nielsen [75] suggested, the only action for removing pollution is the diluting process. It can be applied with outdoor ventilation air, that is, generally, the main method for maintaining adequate IAQ in buildings. In this direction, Chuaybamroong et al. [76] indicated that high ventilation rate can dilute the levels of airborne microbes, but taking care of environmental (energy consumption), social (thermal discomfort for users), and economic (maintenance costs for oversized HVAC (heating, ventilation, and air conditioning)) sustainability of the facilities [77–79].

In conclusion, as Salonen et al. [23] suggested proper and efficient ventilation combined with low-emission building materials that can be key factors for an adequate IAQ and control of infections spread by air. Odors may trigger strong negative physical reactions in some users, as the analysis by Prior et al. [80] demonstrated that the odor of dimethyl sulphoxide (DMSO) in hospital settings gave rise to headaches and gastrointestinal reactions, especially for hospital staff (nurses).

For high performances, according to Hoseinzadeh et al. [81], it is suggested that hospital managers attempt to qualitatively and quantitatively evaluate indoor air of hospitals periodically, and they should place and use air purification equipment in hospitals during design and construction phases.

### 5.3. Maintenance and Operational Strategies

The relationship between bioaerosol and chemical pollution and ventilation, maintenance, and cleaning activity is fundamental because they could play an important role in pollutant concentrations in hospitals [82].

Innovations in medicine field and ancillary technologies affect the need to upgrade air conditioning systems and to verify hospital microclimatic conditions. The scientific community has highlighted the strategic role of ventilation in healing environments and it is well known that an appropriate air conditioning is useful for prevention not only of several communicable diseases but also in the reduction or elimination of the spread of physical and chemical contaminants, as long as the maintenance and cleaning are regular [30]. In fact, as several scholars highlighted, infection control issues involve also fungal or bacterial contamination within hospital settings [13]. If ventilation systems are properly designed, installed, maintained, and kept clean to preserve the correct pressure between several healing environments, it is possible to reduce airborne diseases.

On the contrary, inadequate maintenance of HVAC systems can cause microbial growth through bacterial and mold spores collected in air filters, as Simmons et al. [83] have demonstrated in an analysis on seven hospitals in the eastern United States.

Another aspect to be considered is the procedures related to the maintenance activities: the removal of ceiling tiles, or similar finishing materials, to verify mechanical and electrical equipment, without any precautions to minimize dust emissions or occupant exposure to particles, can be dangerous for the health status of users [35, 84]. This activity had a significant impact on fluorescent particle concentration because the airspace cavity overlying the suspended ceiling accumulates contaminants, molds, biofilms, etc. [85].

The maintenance activities correlated to the replacement of finishing materials require a selection of high quality materials with adequate performances; in fact, as Anderson et al. [86] reported in a hematology ward, any airborne *Aspergillus fumigatus* was not confined in the ceiling plenum by the monitoring, but airborne disease was caused by the insulating material in ceiling tiles.

For this reason, it is fundamental to control the furniture and material selection. Kildesø et al. [87] studied the release of particles from indoor fungi growing in building materials, in particular in wetted wallpapered gypsum boards and on the surface of ceiling tiles.

## 6. Design Factors

### 6.1. Dimensional Aspects, Room Configurations, and Door Motion

The scientific literature currently does not provide many references in relation to dimensional aspects and room configurations of an inpatient room. The existing references are defined by the literature for the relationship between surface per bed and the minimum requirements to be fulfilled, referring to regulations. In addition, there are not any specific guidelines in inpatient room's dimensions, solar exposure strategies, and opening dimensions, in relation to IAQ. In order to investigate the dimensional aspects and rooms, the authors have investigated several case studies in international scenario that show several configurations of indoor air performances.

Several case studies and simulations have been carried out for understanding the air fluxes and the correlation among users, ventilation systems, room dimensions, etc., but they do not define any specific dimensional requirement and/or optimal room dimension for inpatient wards. Only one suggestion by Escombe et al. [88], who reviewed the volume of isolation rooms in relation to potential risks, stated that room should have a volume equal to 31 m^3^.

In addition, it is necessary to underline the fact that simulations in empirical studies can give rise to design and operative strategies but they cannot be considered as real outcomes because they have not been applied in real contexts. Bivolarova et al. [39] suggested that there are many differences between the tracer gas and aerosol particles. As Tang et al. [89] reported, airborne particles smaller than 5–10 *μ*m can be simulated with tracer gas, since they often stay suspended in the air for long time. The scholars suggested that the particles follow the air stream, although only a few research studies have compared particle movement and tracer gas and in ventilated rooms. All the simulations and studies are listed in Table 3.

### 6.2. Door Motion

Another aspect that can influence the IAQ is related to door motion. In fact, the volume exchanged between two spaces due to door motion can affect the indoor air in relation to air fluxes.

Kiel and Wilson [100] developed several analyses to determine the fluid volume that is exchanged through an external doorway. Their research studies were based on densimetric Froude number. Since the experiment mainly concerned the buoyancy-induced flow, they realized models and full scale measurements with tracer gas. At a density difference between rooms equal to zero, the scholars demonstrated that exchange volume grows linearly with door opening speed. In addition, their studies highlighted that the volume exchanged was almost constant with the hold open time (maximum opening angle).

Moreover, Eames et al. [92] demonstrated, supported by isothermal flow in relation to several door opening angles, that exchanged volume varies around 1.5% and 5% of the entire room volume and it is comparable to the volume swept by the door.

Afterwards Kalliomäki et al. [101] quantified the relationship between door opening times and hold open times on the exchanged volume. The analysis demonstrated that exchanged volume increases with hold open time and total cycle time (opening, hold open, and closure), while it does not vary with door speed for a certain hold open time, as Hathway et al. [94] highlighted.

In addition, as several authors demonstrated, door motion is related to the movement of users through the doorway, which can also contribute to the air exchange. As Tang et al. [102] highlighted, walking through a doorway, there is flow both ways, into and out of the room. In addition, Kalliomäki et al. [103] analyzed the volume of air exchanged between rooms from only one door opening and door opening and manikin movement combined, and they demonstrated that there is a slightly greater exchange when manikin movement is considered (approximately one quarter).

In conclusion, starting from the simulations and analysis by the scholars, Kalliomäki et al. [103] investigated the influences related to sliding or hinged door. With a temperature difference of 2°C, the values grow by 303% for the sliding door and 41% for the hinged one: in fact, on the basis of tracer gas measurements, the research project demonstrated the exchange volume ranged between 0.3 m^3^ and 2.3 m^3^ for sliding door and 1.2 m^3^/2.4 m^3^ for hinged one, data highly affected by parameters imposed. In each case study, it was clear that the exchange volume was significantly lower for sliding door than hinged one (although the air exchange between the isolation room and its anteroom increased notably while increasing the hold open, total cycle, and opening times with both door types [103]).

### 6.3. Finishing Materials and Furniture

Although the deterioration of indoor air cannot be attributed to a single cause, another factor is related to materials and furniture. This is the reason why designers have a great responsibility in IAQ: localization and orientation of the buildings, the choice of appropriate technical solutions, responding to the regulations, strategies for reduced building energy consumption, use of healthy materials in interior design, etc. [104].

Building materials, finishing, and furniture containing VOCs include resilient floorings, rugs, walls covering, fabrics, furniture, ceiling tiles, composite wood products, insulation, paintings and coatings, adhesives, stains, sealants, and varnishes. Typically, formaldehyde is used in composite wood and batt insulation, as well as in manufacturing process to protect fabric against shrinking, for improved crinkle resistance, dimensional stability, and color fastness. Zuraimi et al. [67] sustained that building decorations are important sources of TVOCs and formaldehyde in indoor spaces. As Oberti [105] studied, in some cases, it is used for finishing treatments for improving stain resistance.

These components are absorbed in high quantities and they are subsequently released in different ways during the time: the harmful substances are usually released in large quantities as soon as they are applied and over time they tend to decrease; in fact, they are also used to detection and sampling depending on the materials adopted.

These substances can affect health status of individuals, and the harmful effects range from sensory discomfort to severe health impairment, at high concentrations in indoor environments. Some of them are recognized as carcinogenic such as benzene, carbon tetrachloride, chloroform, trichloroethylene, tetrachloroethylene, etc.

As Tucker [106] demonstrated, VOCs emitted high levels of compounds when a product is installed and its emissions diminish gradually over time. Semivolatile organic compounds are used in building materials to afford flexibility, stain repellence, water resistance, etc. [107]. Among them, there are phthalates in PVC, building products, upholstery, wall coverings, hospital and shower curtains, etc., but they are used also in nonbuilding materials, such as medical devices [108]. In conclusion, perfluorochemicals are used in carpets, upholstery, textiles, and furniture; in other cases halogenated flame retardants are used for furniture, electronic equipment and foam pillows, etc (when stain resistance or water repellence is required).

Other affecting factors are physical contaminants, such as fibrous insulations, under certain stress conditions, which could be responsible for releasing mineral fibers into the air, so much more dangerous to health both because their diameter is shorter and more easily breathable [109]. In addition, heavy metals are used as stabilizers in vinyl plastic materials, and they can be found in a variety of other uses in roofing, solder, radiation shielding, and in dyes for paints and textiles. Heavy metals can be considered dangerous: belonging to the group of metallic elements, they can be highly toxic, such as arsenic, antimony, cadmium, chromium, copper, cobalt, lead, mercury, and zinc.

Albeit less frequent in healthcare environments, many materials used during construction phase and in finishing materials can emit radon [110].

Moreover, recently, several companies are giving rise to materials with noble metal nanoparticles. Titanium dioxide (TiO_2_) is one of the most popular photocatalysts to reduce VOC pollutants in air, and it works with UV light (≤387 nm), which oxidizes molecules [111]. They are technologies not well known for their limited absorption of the visible light (400–700 nm) [112]; the use of these noble metals increases titanium dioxide activity in the visible light range.

In conclusion, as several scholars sustain, material selection could affect user comfort and well-being [23]. As Ulrich [113] suggested, comparisons of the advantages for patients of different types, for example, of flooring materials, including carpet and hard or glossy materials (linoleum, vinyl composition, etc.), have found increasing suggestions that carpet is better from the standpoint of certain user-centered considerations. For this reason, as a consequence, it is necessary to apply appropriate cleaning strategies [114].

In the next paragraphs, some specific considerations around some materials are analyzed and tested by several international scholars.

### 6.4. Flooring

In healthcare facilities, it is requested to respond to hygienic requirements. Floors and walls need to be durable and easy to clean. Currently there are several design strategies.

Among the finishing materials for floors and walls, linoleum is a material composed entirely of natural raw materials (wood flour, cork flour, flaxseed oil, jute, and natural resins) and which requires the installation of solvent-free adhesives [115]. It is the most suitable material for use in inpatient rooms. Linoleum, such as rubber and PVC, responds to the safety and hygienic requirements for healthcare settings; however, it differs for the naturalness of the components.

This material is applied directly on the floor using special bonding products. In addition, it is among the most harmless vinyl adhesives in water dispersion.

Once the linoleum is laid, the final surface is obtained by spreading a specific metallic wax. This process can be considered a disadvantage, as this operation must be repeated periodically throughout the life cycle of the material [115].

In the case of scratching or wear of the surface layer, the lower layer is hardened by restoring the surface qualities of the coating and by smoothing the irregularities of the surface through the linseed oil that in contact with the air absorbing oxygen it cures and increases the volume. In general, the use of this material is recommended for inpatient room but it needs a regular maintenance during the years.

Another material for an easy cleaning and maintenance, and for its durability and safety features, is PVC. However, although it has not a natural composition, this material releases a wide range of VOCs, including plasticizers and solvent residues. In addition to the emissions due to its composition, the adhesives used for the commissioning are one of the main sources of VOC emissions. PVC coating products on the market vary considerably. Therefore, it is necessary to consider the specific features of these products [105].

Generally, it is well known that carpets and rugs in healing environments are not recommended for bacteria and dusts' accumulation, and they are not fast to clean. According to Kemper et al. [116], microbial colonization in indoor environments, especially carpeting, is well known, and several studies have showed that a variety of fungi and bacteria can be aerosolized from these microbial reservoirs [117]. In other words, carpets generally cover expansive horizontal surfaces and they are accumulators of harmful microbes both settling airborne particles and then in the air [35].

Some studies conducted in the hospital rooms have confirmed that issue. Pereira et al. [35] and Bhangar et al. [118] investigated the concentration of total fluorescent particles of carpeting, respectively, in hospital environments and office facilities demonstrating that the proportion of fluorescent in total airborne particle concentration increased during walking, in comparison to the times when the room was unoccupied. Previously, also Anderson et al. [119] associated high levels of microbial contamination with rugs in inpatient rooms. The microbiological and epidemiological investigation permitted to analyze the hospital rooms, with and without carpets.

In conclusion, currently there are some innovations in healing environments with the introduction of materials with bactericidal proprieties, self-cleaning ones, antiodor efficiency and effectiveness against nitrogen oxides (NOx), etc., in particular, as highlighted in [120, 121]: (a) bactericidal effect through the oxidizing power of photocatalytic action. It is possible to remove the bacteria (photocatalysis does not really kill the bacteria but it decomposes them, irretrievably damaging their cells and causing their death); (b) self-cleaning properties through the photocatalytic activity. It is possible to guarantee a dual effect on the daily dirt on the floors and walls (powder, organic residues, etc.); (c) antiodor efficiency permits to degrade the most common organic molecules that cause the smell; (d) effectiveness against NOx through the photocatalytic process (i.e., titanium dioxide deposited). It is possible to destroy and transform a lot of pollutants and toxic substances in harmless compounds such as nitrates, sulfates, and carbonates. Currently, some companies have introduced materials (such as porcelain grés tiles) with titanium dioxide.

### 6.5. Paintings

There are numerous nontoxic paints, with zero or low VOC emissions. The current standards define that a paint can be labeled with low VOCs if it is lower than 250 g/l, while those with zero VOCs must respond to a value lower than 5 g/l. In any case, the release of VOCs, despite the use of a low or zero release paint, depends on the amount of product used and the painted surface.

However, the VOC content of paints is generally evaluated only for the base paint [122]. Coloring can significantly increase potential emissions, so it is advisable to request data emissions for the formulation of the final paint that will be applied.

The United States Environmental Protection Agency (US EPA) provides guidelines for applying indoor environments that can also be used for hospital settings: (a) painting activity with the absence of users; (b) natural ventilation (and/or regular ventilation) for the environments during and after painting.

Recent and innovative solution in paintings is the integration with silver particles. As Kyung-Hwan et al. [123] stated, silver at nanolevels has a great antimicrobial property (99%), as well as antifungal and antiviral effects.

Naddafi et al. [122] have investigated the performance of ordinary and nanosilver paintings in some hospital rooms in Tehran University of Medical Sciences. The analysis was applied on some rooms in an infectious diseases unit with the same environmental features (two beds with patients and frequency of cleaning activities, temperature, day-lighting, ventilation conditions, etc.). One of the healing spaces was painted with ordinary paint, and the other two rooms with two different nanosilver paints (2%) provided from two companies to examine the effect of nanosilver paintings on decreasing the microbial burden in indoor air. For understanding possible effects to the patients, the samplers were located with a distance of about 1.2 m to 1.5 m from the patient's breathing zone. The results, from the biological point of view, showed that both nanosilver paintings had no statistically effects on burden of bacterial contamination; on the contrary, the sampling method did not permit to assess the ordinary paint.

Although the results are positive, Kaiser et al. [124] stated that paint industries consider the use of nanosilver, as well as photocatalytic active nanotitanium dioxide or nanosilica dioxide as additives for the protection of indoor and outdoor surfaces, against physical, chemical, and microbial deterioration, as alternative to conventional additives. Currently, there are not any scientific demonstrations if nanoparticles in paintings will achieve the proposed effects during the time and the potential risks for environment and human health.

### 6.6. Wood

Natural materials have a positive influence on the well-being, although there is not widespread in healing spaces for hygienic conditions [125]. As Bringslimark [126] demonstrated, there are beneficial outcomes of indoor natural elements by hospital users. As Kirkeskov et al. [127] analyzed, wood contains numerous other organic and inorganic compounds.

Analysis by Kirkeskov et al. [127, 128] investigated the chemical concentrations in wood products. Ten types of wood imported in Denmark, product groups widely used, were monitored. Typically, these products undergo a surface treatment, which can either contribute to compounds' emission or reduce the emission from the wood by creating an impermeable sealing [128]. For the investigation, it was used a method able to carry out a toxicological evaluation of compounds emitted from wood and wood-based materials, furniture, and interior furnishings, developed by Jensen et al. [127]. The analysis was applied on some inpatient rooms with specific thermohygrometric conditions [128]. The results obtained, based on random samplings, registered low concentrations of 25 chemical compounds.

Several studies have confirmed that untreated wood species have the same low emission rates, as confirmed by Jensen et al. [127] and Nyrud et al. [129]. The last ones have done a deep monitoring for quantifying possible beneficial psychological effects of wood use and measuring the effect of exposure to different wood interiors in the healing process. The analysis considered eight inpatient rooms with different wood panels. During the investigations, temperature and relative humidity were monitored continuously. Neither of the parameters have any significant variation, so the HVAC system guaranteed good performances. The final outcomes reported a range between 115 and 170 ng/l, values significantly lower than newly furnished buildings [129]. Starting from the results, it is possible to suggest wood finishing materials in inpatient rooms do not have any substantial effects on indoor air.

In general, the emission of chemical compounds from the products is very low. This could be due to several reasons, as Kirkeskov et al. [128] listed: (a) during drying, many compounds are emitted; (b) during processing, further emission takes place and some of the surface treatment will contribute to encapsulating the compounds in the wood, thereby limiting emission; (c) also transportation permits to disperse further emission of chemical compounds. In conclusion, the measurements and toxicological evaluations highlight that emissions of individual compounds from the examined wood species or their surface treatments are not expected to cause adverse health effects.

## 7. Human Presence and Medical Activities

### 7.1. The Role of Human Behavior and the Influence of Human Occupancy

As several research projects have already stated, the main sources of indoor bioaerosols in healthcare settings include users [130]. According to Cairns et al. [131], hospital staff, patients, and visitors increase the contamination of airborne pathogens within the inpatient ward. As Scheff et al. [132] evidenced, high particle levels can be associated with human activities, air change rate, and filtration efficiency.

In addition, users carry particles and spread them. Bhangar et al. [118] stated that human occupants are an important source of microbes in indoor environments in two ways: one by resuspension from room surfaces, whereby occupants' movements disturb microbial materials that had previously settled onto or colonized indoor materials; the other by direct shedding of particles from bodies and clothing [133]. Regarding the latter, Tormo-Molina et al. [60] demonstrated that airborne and pollen distribution in healthcare facilities can be caused not only by natural or mechanical ventilation systems but also by humans who, as vectors, can transport airborne pathogens through their clothes or stirring them up from the floor where they are abundant, as people walk inside a building [134].

In support of these considerations, among the studies, Gulshan et al. [59] in Sheikh Zayed Hospital, a tertiary care hospital located in Lahore (Punjab Pakistan), have studied contributions of indoor and outdoor sources to PM_2.5_ in different inpatient wards (medical, pulmonology, surgical, pediatrics, and nephrology). The wards have different geometries in their layouts, indoor particle sources, outdoor environments, and ventilation patterns.

The study evidences that although the hospital is centrally air conditioned, the wards are not airtight thereby allowing infiltration from the outside mainly by doors. In average, the highest levels of PM_2.5_ occurred during doctor's visit, visiting hours, breakfast/dinner time, and prayer time. Also Tang et al. [135] highlighted that particle mass increased significantly during visiting hours in a Taiwanese hospital.

In addition, Pereira et al. [35] verified in some hospitals that the peaks of particle levels are influenced by the number of people per unit area and the main human activities are washing hands in the sink, bed making, the use of medical sprays, plant room activity, nebulization therapy, cleaning activities, etc. It is evident the linear relationship exists among (a) particle concentrations, (b) carbon dioxide concentration, and (c) the number of occupants, as Tang et al. [130] and Quadros et al. [136] demonstrated.

In conclusion, starting from the scientific literature and, in particular, from Sudharsanam et al. [137], in relation to nature of the movements, the intensity of human activities can be subdivided into: (a) minimum as talking; (b) moderate as movement of patients and delivering healthcare to patients; (c) maximum as movement of patients, delivering healthcare to patients, and cleaning activities, which includes bed making and floor and furniture cleaning.

### 7.2. Human Health Status

Some recent studies demonstrated that health status of users can affect the indoor air. In fact, although at the beginning of medicine, physicians diagnosed some diseases through the smell of human breath, in recent decades breath analysis demonstrated the presence of hundreds of volatile compounds in human breath [138, 139]. Since then, up to 3000 VOCs have been identified.

As Buszewski et al. [140] stated, the main constituents of human breath are related to nitrogen (∼73%), oxygen (∼16%), water (relative humidity) (∼5%), and carbon dioxide (∼5%). In addition, less than 1% is composed by minor volatile compounds such as hydrogen, carbon monoxide, ammonia, acetone, methanol, ethanol, propanol, and acetaldehyde [138].

As Morisco et al. [141] stated, liver plays a key role in metabolism and the ingested food, and it can give rise to endogenous volatile compounds. As some studies demonstrated, the influences due to smoking, using mouthwash, brushing teeth, drinking alcohol and coffee, and consuming foods containing garlic, onion, mint, and similar flavored meals can affect the production of air pollution, and several studies analyzed the exhaled breath by patients with lung cancer [142].

Currently, there are several tools for breath analysis, but they do not assess the temporal changes of VOCs in fast processes.

### 7.3. Medical Activities

Starting from the systematic review, several studies have highlighted the influence of some specific medical activities in quality of indoor air.

An emblematic issue is related to radon. It is well known that breathing high concentrations of radon can cause lung cancer and therefore, designers should adopt design strategies (in basements and material selection) for preventing possible adverse effects on health [143]. Recent studies in medical field, as Śmiełowska et al. [71] highlighted, have demonstrated that (in rare cases) radon is used for punctual medical treatments for cancer [144] and also to treat other diseases, such as radon baths to prevent autoimmune diseases such as arthritis, endocrine disorders, etc. [145]. Starting from this scenario, radon has to be considered a significant xenobiotic that might affect the IAQ.

Pereira et al. [35], analyzing sources and dynamics of fluorescent particles in hospitals, have noted that nebulization therapy is a strong source of increases in pollution concentration. These findings correspond to the studies by Roberts et al. [146] in which the authors demonstrated large microbiological peaks occurrence during a period of nebulization (nebulization therapy refers to the delivery of a drug to the body in an aerosolized form via the airways [147]). It is important to observe that the respiration during the therapy could not be decoupled from the particles generated by the nebulizer itself.

Other risk for users is moxa smoke, as Lu et al. [148] evidenced. Moxa smoke has disinfectant and antibacterial properties and causes adverse reactions whose symptoms are similar to those of hay fever [149, 150]. Several studies have reported that high concentrations of monoaromatic hydrocarbons (MAHC) and polycyclic aromatic hydrocarbons (PAHs) have a great impact on health status in moxibustion rooms. Moreover, Hsu et al. [151] have analyzed aldehyde levels during moxibustion in a Chinese medicine clinic, demonstrating mean formaldehyde and acetaldehyde concentrations dangerous for medical staff and patients. As a strategy, the authors have demonstrated that air quality can be improved by ventilation.

Another pollutant concentration is related to nitrogen dioxide (NO_2_), which might be due to anesthetics and medicines (as well as smoke from cigarettes by irresponsible users and workers) [152]. Excessive exposure can cause adverse effects to health status such as decreasing lung function and increasing the risk of respiratory symptoms, etc.

In conclusion, as Sattler and Hall [153] suggested that hospital staff have a very strategic role in hospital settings. They can guarantee environmentally healthy and safe places for patients and visitors, following environmentally conscious waste management strategies, decrease the use of chemical pollutants, promote the use of healthy foods, provide leadership in environmental stewardship, etc.

### 7.4. Medical Equipment

Among the medical equipment, Śmiełowska et al. [71] highlighted an emerging issue in indoor air: the presence of endocrine disruptor chemicals (EDCs) that influence reproduction. They include phthalates, which can be emitted by plastifiers and polymer materials, such as plastic infusion bags, blood bags, plastic film, injectors, rubber tubing, etc. As Wang et al. [108] stated, phthalate concentrations are higher in pharmacies and transfusion rooms, due to the features of equipment of rooms and workstations.

As a consequence, medical equipment can be a potential source of phthalate emissions into the air, especially because they are localized near the breathing zone of patient when they are used.

### 7.5. Anesthetic Gases

It is well known that anesthetic gases are monitored in hospital settings. However, in general, in the inpatient room, anesthetic gases are nor required; some case studies have gas installation in headwall, and therefore, several suggestions have been listed.

Anesthetic gases are gaseous pollutants in healthcare facilities. Typically they are halothane, isoflurane, sevoflurane, and nitrous oxide [15], but they also include formaldehyde, ethylene oxide, and glutaraldehyde. Each of these substances is toxic if it occurs at high concentrations, and longer exposures can cause several health effects, such as asthma, dyspnea, chest pains, and irritations [71]. In particular, ethylene oxide and formaldehyde, at high concentrations in the air, have carcinogenic and mutagenic properties [154, 155]. Moreover, as Śmiełowska et al. [71] suggested that chronic exposure to such pollutants can cause severe damage to the liver and kidneys, as well as it becomes harmful to pregnant women as it may cause miscarriages and congenital defects to fetus.

The existing extracting systems ensure to reduce these gases with low occupational exposure and negligible release into the environment, although typically their application is in operating rooms.

### 7.6. Future Perspectives

Currently, several studies are considering the introduction of personal wearable pollution devices [156] that embed some sensors for environmental parameters, cardio and respiratory signals of users, pollution concentrations (as the pump test), etc. [157]. New frontiers can consider personal devices, near the breathing zone of the user, that assess chemical and biological pollutant levels.

## 8. Conclusions

The systematic review gives rise to a broad scenario on the existing knowledge regarding the indoor air pollution and design and management strategies. Although the aim of the investigation was strictly related to chemical pollution, several considerations from the biological point of view have been listed.

It highlights the focus on several pollutant VOCs (acetaldehyde, benzene, ethylbenzene, toluene, xylene, etc.), anesthetic gases (halothane, nitrous oxide, sevoflurane, etc.), and other compounds (PM_2.5_ and PM_10_, polycyclic aromatic hydrocarbons, carbon dioxide, carbon monoxide, monoaromatic hydrocarbons, nitrogen dioxide, etc.), but it is clear that there is a huge shortage on the topic without specific data analysis and data comparison.

As emerged from the analysis of several factors, to overcome the criticisms related to the design of healing environments, interdisciplinary knowledge needs to be taken into account: (a) the needs of users (hospital staff, patients, outpatients, visitors, etc.) related to their activities and therapies; (b) needs and problems related to users in relation to nosocomial infections; (c) applications of the technologies and operating systems needed to carry out the ordinary and specialist healthcare disciplines; (d) risk analysis techniques to several functional units, including events caused by incorrect application of procedures; (e) acceptable residual risk values and related sharing and management procedures [17].

The systematic review reveals a lack of specific protocols related to chemical pollution related to cleaning activities; although several guidelines by green public procurements define some specific prescriptions, the concentration levels of cleaning products can affect the performances of air.

In addition, designers, in collaboration with healthcare professionals, should (a) design the healthcare settings according to the different uses; (b) support the healthcare organization to identify the most optimal solutions, both for the technical, functional, economic, and management aspects; (c) elaborate and monitor the management and maintenance procedures of environments and systems; (d) train staff who will use or manage the spaces and systems, through the processing and updating of appropriate procedures, training courses, and monitoring; (e) select material strictly correlated to the cleaning products and activities.

Exposure of hospital users to chemical pollutions is related to several aspects due to product formulations, activities and procedures, the inexperience of sanitary and not sanitary staff, etc (Bessonneau et. al., [48]).

The systematic review, supported by the existing scientific literature, becomes a starting point for considering the importance of the topic and to stimulate the knowledge around this issue for improving studies, analysis, simulations, monitoring activities, etc. Detailed systematic reviews on each field of interest should be done for specific analysis and punctual outcomes and data analysis.

As the analysis highlighted, the research field should be more and more explored through monitorings and assessments of exposure concentrations. As Bessonneau et al. [48] observed, data have to be confirmed in a multicentric approach, and research efforts must be designed with regard to the possible health effects induced after inhalation exposure to a complex mixture of chemical compounds.

Currently, the authors are developing a research group which monitors the activity of air quality in inpatient ward, focusing on chemical pollution for understanding the current values and room features, maintenance activities, and medical procedures, with a replicable protocol for a broad investigation in the EU [158], supported by ISO 16000 and studies by Istituto Superiore di Sanità (Italian National Institute of Health). The aim of the research project is to define design, management, and operative strategies for supporting healthcare organizations and avoiding possible deterioration of users' health status.

## Figures and Tables

**Table 1:** General overview of the existing norms and guidelines related to indoor air.

Norms and guidelines		Aims and scope	Relationship with healing spaces	Contents
UNI 10339:1995 Italy	Aeraulic systems for users' well-being and comfort	References for microclimate and ventilation requirements in some functional units	References for limited functional units	(i) Microclimatic requirements (ii) Ventilation requirements
DPR 14/01/1997 Italy	Act for the minimum structural, technological, and organizational requirements for public and private healthcare facilities	References for microclimate and ventilation requirements in some functional units	References for limited functional units	(i) Microclimatic requirements (ii) Ventilation requirements
ISPESL (2005 and 2009) Italy	Standard guidelines safety and hygiene in surgery block, emergency room, and children block	References for safety and healthy hard functional units	References for limited functional units	(i) Microclimatic requirements (ii) Ventilation requirements
Legislative decree 81/2008 Italy	Law on health and safety in working places	References for workers safety (generic for several workspaces)	Any reference related to hospital	(i) Guideline values
ASHRAE 170/2008 USA	Ventilation of healthcare facilities	References related to ventilation requirements for all the hospitals	References for several hospital functional units	(i) Ventilation requirements
WHO 2006	WHO air quality guidelines for particulate matter, ozone, nitrogen dioxide, and sulfur dioxide	References related to chemical pollution for ambient air	Any reference related to hospital	(i) Guideline values
WHO 2010	WHO guidelines for indoor air quality: selected pollutants	References related to chemical pollution for indoor	Any reference related to hospital	(i) Guideline values
WHO 2016	Ambient air pollution: a global assessment of exposure and burden of disease	References related to chemical pollution for outdoor	Any reference related to hospital	(i) Guideline values (outdoor)
ISO 16000	Air in confined environments	References related to sampling methods	Any reference related to hospital	(i) Sampling strategies

**Table 2:** Indoor air factors in inpatient room.

Criterion	Field of interest	Influence	Focus
Design factors	They refer to all the components that characterize the inpatient room (room dimensions, furniture, finishing, etc.)	Their emissions are constant, although in relation to their life, the emissions may decrease over the time	(i) Dimensional aspects, room configuration, and door motion (ii) Finishing materials and furniture
Management and cleaning activities	They refer to the management and maintenance activities, ventilation systems, cleaning and disinfectant activities, etc., carried out in the room and in the functional units	They can highly affect the indoor air, but their emissions can be controlled through the applications of strategies, and in the same time, they can be changed if their actions are dangerous for users	(i) Cleaning and maintenance activities (ii) Ventilation systems (iii) Maintenance and operational strategies
Human presence and activities	They refer to the presence of users, their health status, and the medical activities carried out in the inpatient room	Their presence and application can vary, and therefore they can affect the indoor air in different modes. In general this component does not affect highly the indoor air performances	(i) Human behavior (ii) Medical activities (iii) Medical equipment
Outdoor and microclimatic factors	They refer to the outdoor air, the solar exposure, and microclimatic parameters	Although these factors can vary, they have a great influence on the indoor air and the performances of materials in the room and air fluxes	(i) Outdoor issues and site localization (ii) Microclimatic parameters (iii) Solar exposure

**Table 3:** List of references related simulation analysis.

Reference	Topic
Bivolarova et al. [39]	Simulation of air distribution in inpatient rooms
Bolashikov et al. [77]	Simulation of exposure to exhaled air from sick occupant with wearable personal exhaust unit
Chen et al. [90]	Simulations and effects due to temperature difference in indoor air performances
Devlin [91]	Simulation of an active chilled beam design
Eames et al. [92]	Simulation of movement of airborne contaminants
Emmerich et al. [93]	Simulations of strategies to reduce the spread of airborne infectious agents
Hathway et al. [94]	Simulations of air exchange due to hinged-door motion
Memarzadeh [95]	Simulation of strategy to control aerosol-transmitted infections in a hospital suite
Nielsen [65]	Simulations of airborne infectious diseases
Olmedo et al. [96]	Simulation of airborne cross infection with vertical low-velocity ventilation
Qian et al. [9, 97]	Simulations in dispersion of exhalation pollutants by manikins
Wu et al. [98]	Simulation of air distribution in inpatient rooms
Zhai et al. [99]	Experimental verification of tracking algorithm for dynamically releasing single indoor contaminant

## Data Availability

The data used to support the findings of this study are included within the article and the reference list.
